# Increased Expression of HCN Channels in the Ventricular Myocardium Contributes to Enhanced Arrhythmicity in Mouse Failing Hearts

**DOI:** 10.1161/JAHA.113.000150

**Published:** 2013-06-21

**Authors:** Yoshihiro Kuwabara, Koichiro Kuwahara, Makoto Takano, Hideyuki Kinoshita, Yuji Arai, Shinji Yasuno, Yasuaki Nakagawa, Sachiyo Igata, Satoru Usami, Takeya Minami, Yuko Yamada, Kazuhiro Nakao, Chinatsu Yamada, Junko Shibata, Toshio Nishikimi, Kenji Ueshima, Kazuwa Nakao

**Affiliations:** 1 Department of Medicine and Clinical Science, Kyoto University Graduate School of Medicine, Kyoto, Japan (Y.K., K.K., H.K., Y.N., S.U., T.M., Y.Y., K.N., C.Y., J.S., T.N., K.N.); 2 Department of Physiology, Kurume University School of Medicine, Japan (M.T., S.I.); 3 Department of Bioscience, National Cerebral and Cardiovascular Center Research Institute, Japan (Y.A.); 4 EBM Research Center, Kyoto University Graduate School of Medicine, Kyoto, Japan (S.Y., K.U.)

**Keywords:** arrhythmia, HCN channel, heart failure, ion channels

## Abstract

**Background:**

The efficacy of pharmacological interventions to prevent sudden arrhythmic death in patients with chronic heart failure remains limited. Evidence now suggests increased ventricular expression of hyperpolarization‐activated cation (HCN) channels in hypertrophied and failing hearts contributes to their arrythmicity. Still, the role of induced HCN channel expression in the enhanced arrhythmicity associated with heart failure and the capacity of HCN channel blockade to prevent lethal arrhythmias remains undetermined.

**Methods and Results:**

We examined the effects of ivabradine, a specific HCN channel blocker, on survival and arrhythmicity in transgenic mice (dnNRSF‐Tg) expressing a cardiac‐specific dominant‐negative form of neuron‐restrictive silencer factor, a useful mouse model of dilated cardiomyopathy leading to sudden death. Ivabradine (7 mg/kg per day orally) significantly reduced ventricular tachyarrhythmias and improved survival among dnNRSF‐Tg mice while having no significant effect on heart rate or cardiac structure or function. Ivabradine most likely prevented the increase in automaticity otherwise seen in dnNRSF‐Tg ventricular myocytes. Moreover, cardiac‐specific overexpression of HCN2 in mice (HCN2‐Tg) made hearts highly susceptible to arrhythmias induced by chronic β‐adrenergic stimulation. Indeed, ventricular myocytes isolated from HCN2‐Tg mice were highly susceptible to β‐adrenergic stimulation‐induced abnormal automaticity, which was inhibited by ivabradine.

**Conclusions:**

HCN channel blockade by ivabradine reduces lethal arrhythmias associated with dilated cardiomyopathy in mice. Conversely, cardiac‐specific overexpression of HCN2 channels increases arrhythmogenicity of β‐adrenergic stimulation. Our findings demonstrate the contribution of HCN channels to the increased arrhythmicity seen in failing hearts and suggest HCN channel blockade is a potentially useful approach to preventing sudden death in patients with heart failure.

## Introduction

Despite recent progress, the efficacy of available pharmacological interventions aimed at preventing lethal arrhythmias associated with chronic heart failure remains limited. Indeed, as many as 50% of deaths among heart failure patients are sudden and unexpected, presumably caused by lethal arrhythmias.^1^ Thus, identification of potential therapeutic targets based on knowledge of the molecular mechanism underlying the enhanced arrhythmicity in failing hearts would be highly desirable.

Hyperpolarization‐activated cyclic nucleotide‐gated (HCN) channels comprise an ion channel family (HCN1‐4) that carries a current termed I_f_ or I_h_, which has been recorded in both the heart and nervous system.^2–3^ In the healthy adult heart, HCN channels are predominantly expressed in the conduction system, especially in the sinoatrial node, where HCN4 is the major isoform and controls cardiac rhythmicity.^3^ HCN channels (HCN1‐4) are also expressed in ventricular myocytes, where HCN2 is the dominant isoform, though expression of HCN channels in the healthy adult ventricular myocardium is generally much weaker than in the conduction system, so that I_f_ currents are rarely detectable in normal ventricular myocytes.^3–5^ During development, HCN channels are abundantly expressed in the embryonic ventricle, but their expression progressively declines after birth, and is restricted to the conduction system in healthy adult hearts.^6^ However, HCN channels, especially HCN2 and HCN4, are re‐expressed in hypertrophied and failing hearts in both rodents and humans, and the resultant increase in I_f_ currents in ventricular myocytes is thought to provide an important trigger that initiates clinically significant arrhythmias in those hearts.^3–7^ Direct evidence showing the contribution of induced ventricular expression of HCN channels to enhanced myocardial arrhythmicity in vivo is lacking, however. Nor has blockade of HCN channel been shown to prevent malignant arrhythmias or sudden death associated with heart failure independently of heart rate reduction.

We recently reported that a transcriptional repressor, neuron‐restrictive silencer factor (NRSF, also named REST) is an important regulator of the fetal cardiac gene program.^8^ Transgenic mice that selectively express a dominant‐negative form of NRSF (dnNRSF) in their hearts (dnNRSF‐Tg) develop progressive cardiomyopathy leading to sudden arrhythmic death beginning at about 8 weeks of age.^9^ Hearts from dnNRSF‐Tg mice show increased expression of fetal type ion channel genes, including *HCN2* and *HCN4*, which is consistent with what has been observed in other animal models of heart disease and in human failing hearts.^6–7,6–11^ Moreover, I_f_ amplitude was correspondingly increased in ventricular myocytes from dnNRSF‐Tg hearts, which raises the possibility that I_f_ currents in some way contribute to the occurrence of arrhythmias in dnNRSF‐Tg hearts.^9^

To clarify the contribution made by HCN channels to the development of arrhythmias associated with heart failure, and to assess the capacity of HCN channel blockade to prevent malignant arrhythmias, in the present study we tested the effects of ivabradine, a HCN channel blocker, on survival and ventricular arrhythmicity in dnNRSF‐Tg mice. We also generated and analyzed transgenic mice overexpressing HCN2 channel in a cardiac specific manner (HCN2‐Tg). Our findings demonstrate that increased expression of HCN channels contributes to the increased arrhythmicity observed in failing hearts.

## Methods

### Animal Experiments

Beginning at 8 weeks of age, dnNRSF‐Tg mice and control wild‐type (WT) littermates were left untreated (control) or were treated with ivabradine (7 mg/kg per day orally) given in drinking water ad libitum. The dose of ivabradine, which minimally affected heart rate in dnNRSF‐Tg mice, was chosen based to earlier reports and our preliminary studies.^12–13^ We adjusted the concentration of ivabradine dissolved in the water based on the water consumption among each group. Ivabradine was supplied by Servier Laboratories.

A mouse HCN2 cDNA was cloned into the SalI site of pBluescript IIKS(+) plasmid containing the α‐myosin heavy chain (MHC) promoter. The resultant α‐MHC‐HCN2 transgenic construct was then released from the vector backbone by digestion with NotI and purified for injection into the pronucleus of fertilized oocytes harvested from C57BL/6J mice. The surviving embryos were then transferred to the oviducts of pseudopregnant MCH mice. Using an osmotic minipump (Alzet osmotic pumps, Durect Corp) according to the manufacture's protocol, isoproterenol (15 mg/kg per day) or vehicle was subcutaneously administered for 7 days, beginning at 23 weeks of age. The animal care and all experimental protocols were reviewed and approved by the Animal Research Committee at Kyoto University Graduate School of Medicine.

### Patch Clamp Studies

Ventricular myocytes were dispersed as reported previously, and perfused with the physiological bathing solution during the experiment.^9,14^ The physiological bathing solution contained (in mmol/L); 140 NaCl, 5.4 KCl, 0.5 MgCl_2_, 1.8 CaCl_2_, 5 HEPES (pH=7.4 with NaOH). We used Axopatch200B amplifier and Digidata 1320 interface (Axon Instruments, Inc.) for the electrophysiological measurements. The patch pipettes were filled with high K solution containing; 100 K‐Aspartate, 30 KCl, 5 Na_2_‐creatine phosphate, 5 K_2_ATP, 1 MgCl_2_, 5 EGTA, 5 HEPES (pH=7.2 with KOH). We recorded action potential with perforated patch method (0.3 mg/mL amphotericin in high K solution), and I_f_ current with ruptured patch method. A stock solution of 10 mmol/L ivabradine in dimethyl sulfoxide was diluted to the desired concentration using Na^+^‐free bathing solution (final concentration, 3 μmol/L). Since the onset of ivabradine effect is relatively slow, we evaluated the effect of ivabradine during the second minute after addition of ivabradine.^15^

### Intracardiac Electrophysiology

Mice were intubated and anesthetized with 0.5% to 1.5% isoflurane, after which surface electrocardiography leads (limb leads) were placed. Then, using a 1.7 French octapolar catheter (CIBer mouse EP, NuMe, Hopkinton) inserted via the jugular vein, a standard electrophysiological study protocol was performed as described previously.^9,16^ Rapid ventricular pacing using the extrastimulation (S_1_S_2_) technique was carried out using 2 to 3 extra stimuli to determine the ventricular refractory period and to attempt induction of ventricular arrhythmias. The stimulation was administered at twice the ventricular diastolic capture threshold.

### Noninvasive Blood Pressure and Heart Rate Measurements

Systolic blood pressure (SBP) and heart rate (HR) were measured in conscious mice using the tail‐cuff method (Softron Co Ltd) as described previously,^9^ unless otherwise indicated.

### Echocardiographic and Hemodynamic Analysis

Echocardiography was performed using an echocardiography system (Toshiba power vision 8000, Toshiba Corp) equipped with a 12‐MHz imaging transducer as described previously.^9^ Hemodynamic parameters assessed by catheter in WT mice and dnNRSF‐Tg mice were obtained as described previously^9^

### Histological Examination

Hearts were fixed in 10% formalin and prepared for histological analysis as described previously.^9^

### Quantitative RT‐PCR Analysis

Using 50 ng of total RNA prepared from ventricles, levels of mouse *ANP*,* BNP*,* SERCA2*,* CACNA1H*,* HCN2*,* HCN4*,* Col1a1*,* Col3a1*,* FN1*,* MMP2*,* MMP9*, and *Tgfb1* and *GAPDH* mRNA were determined by quantitative real‐time PCR using the manufacture's protocol (Applied Biosystems, Inc) as previously described.^9,17^ The primers and the probe sets were purchased from Applied Biosystems.^9,17^

### Ambulatory Electrocardiography

To monitor ambulatory electrocardiographs, radio frequency transmitters (TA 10ETA‐F20; Data Science) were implanted as previously described.^9^

### Heart Rate Variability

Fifteen‐minute periods of electrocardiographic data with little noise and few ectopic beats were collected 3 to 5 days after implantation of radio frequency transmitters and then analyzed for heart rate variability (HRV). Because of its circadian rhythm, 4 HRV data sets collected during periods extending from 7:00 to 12:00, 13:00 to 18:00, 19:00 to 24:00, and 1:00 to 6:00 hours were averaged for each mouse. Spectral analysis of the heart rate recordings using a fast Fourier transform (FFT) algorithm on sequences of 1024 points was carried out using HEM 3.4 software (Notocord). Cut‐off frequencies for power in the low‐frequency (LF: 0.15 to 1.5 Hz) and high‐frequency (HF: 1.5 to 5.0 Hz) ranges were based on previous experiments with mice.^18^ After FFT analysis, the data that contained ectopic beats or arrhythmic events were deleted manually. In mice, HRV predominantly correlates with parasympathetic activity.^18^

### Statistical Analysis

Survival of dnNRSF‐Tg mice treated with and without ivabradine was monitored for 24 weeks, beginning when the mice were 8 weeks of age. During that period, the numbers of mice that died were recorded, and the survival data were analyzed using the Kaplan–Meier method with the log‐rank test (the outcome was death). We also used nonparametric analyses to evaluate percentage of I_f_ currents, heart rate, blood pressure, body weight, heart weight‐body weight ratio, lung weight‐body weight ratio, hemodynamic parameters, numbers of arrhythmias, numbers of action potentials, water consumption, percentage of collagen area, cardiac gene expression, HRV, and resting membrane potentials. In our analysis of unpaired data using nonparametric tests, the compared groups and conditions were as follows: numbers of arrhythmias in control untreated versus ivabradine‐treated dnNRSF‐Tg mice and in WT versus HCN2‐Tg mice; numbers of action potentials induced by isoproterenol in isolated ventricular myocytes from dnNRSF‐Tg mice treated with vehicle versus ivabradine; the percentage of collagen area in WT versus HCN2‐Tg mice; water consumption by isoproterenol‐treated WT versus HCN2‐Tg mice; cardiac gene expression in WT versus HCN2‐Tg mice; heart rate, blood pressure, body weight, heart weight‐body weight ratio, and lung weight‐body weight ratio in WT versus HCN2‐Tg mice; and hemodynamic parameters in untreated WT versus untreated HCN2‐Tg and in isoproterenol‐treated WT versus isoproterenol‐treated HCN2‐Tg. Comparisons between 2 unpaired groups were made using the Mann–Whitney test. In our analysis of paired data using nonparametric tests, the compared groups and conditions were as follows: the percentage of I_f_ currents in isolated ventricular myocytes from dnNRSF‐Tg and HCN2‐Tg mice recorded in the absence versus presence of ivabradine and the numbers of action potentials induced by isoproterenol in ventricular myocytes isolated from HCN2‐Tg mice in the absence versus presence of ivabradine. Comparisons between 2 paired groups were made using the Wilcoxon signed‐rank test. Comparisons were also made among multiple (more than 2) groups using nonparametric tests. These included: heart rates, blood pressures, body weights, heart weight‐body weight ratios, lung weight‐body weight ratios, hemodynamic parameters, and water consumption among untreated WT, ivabradine‐treated WT, untreated dnNRSF‐Tg, and ivabradine‐treated dnNRSF‐Tg; the percentage of collagen area among untreated WT, untreated dnNRSF‐Tg, and ivabradine‐treated dnNRSF‐Tg; cardiac gene expression among untreated WT, ivabradine‐treated WT, untreated dnNRSF‐Tg, and ivabradine‐treated dnNRSF‐Tg; HRV among untreated WT, untreated dnNRSF‐Tg, and ivabradine‐treated dnNRSF‐Tg; resting membrane potentials among untreated WT, untreated dnNRSF‐Tg, and ivabradine‐treated dnNRSF‐Tg; and heart rates, blood pressures and heart weight‐body weight ratios among control vehicle‐treated WT, isoproterenol‐treated WT, vehicle‐treated HCN2‐Tg, and isoproterenol‐treated HCN2‐Tg. Comparisons among multiple groups were made using Kruskal‐Wallis nonparametric analysis of variance (ANOVA) followed by the Bonferroni correction. Values of *P*<0.05 were considered significant in the experiments other than those analyzed using Kruskal–Wallis nonparametric ANOVA followed by the Bonferroni correction, in which *P*<0.0166 and *P*<0.00833 were considered significant for comparisons among 3 and 4 groups, respectively. Data analyzed by using nonparametric tests are presented as box plots or dot plots.

## Results

### Specific HCN Channel Blocker Ivabradine Improves Survival Among dnNRSF‐Tg Mice

**Figure 1.**
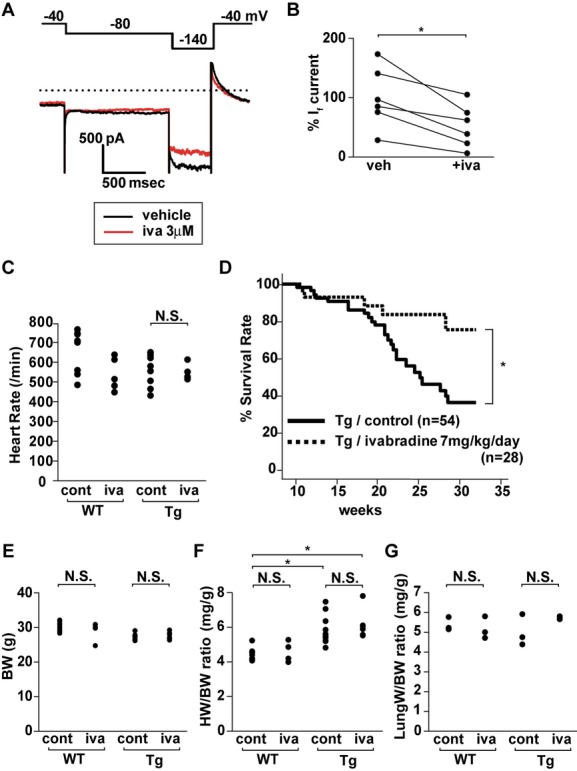
Ivabradine (iva) prolongs survival among dnNRSF‐Tg (Tg) mice. A, Representative I_f_ currents recorded in ventricular myocytes from a Tg mouse in the presence (red line; 2 minutes after the application of iva) and absence of iva (black line). Inward‐rectifier K^+^ current was suppressed by 0.5 mmol/L BaCl_2_. Pulse protocol is shown in the top. B, Relative I_f_ amplitudes (%) measured at −140 mV in the absence (vehicle, indicated as veh) and presence of iva (+iva). A mean relative I_f_ amplitudes (%) in the absence of iva was assigned a value of 100 (n=6 each). *P<0.05 vs vehicle. The Wilcoxon signed‐rank test was used for the analysis. C, Heart rates in wild‐type (WT) and Tg mice at 20 weeks of age, with and without 12 weeks of iva treatment (n=9 for untreated control WT, n=5 for WT with iva, n=10 for untreated control Tg and n=6 for Tg with iva). Kruskal–Wallis nonparametric ANOVA followed by the Bonferroni correction was used for analysis among the 4 groups. NS, not significant. Graphs are shown in dot plots. D, Kaplan–Meyer survival curves for Tg mice, with or without ivabradine. Drug treatment began when the mice were 8 weeks of age and lasted 24 weeks: *P<0.05 (n=54 for Tg without drugs [control], 28 for Tg with ivabradine). E through G, Body weights (BW) (E), heart weight‐to‐body weight ratios (HW/BW) (F) and lung‐to‐body weight ratios (LW/BW) (G) in 20‐week‐old WT and Tg mice, with or without iva (for BW and HW/BW comparisons, n=9 for untreated WT, n=4 for WT treated with iva, n=11 for untreated Tg, and n=6 for Tg treated with iva; for LW/BW comparisons, n=3 in each group). Kruskal–Wallis nonparametric ANOVA followed by the Bonferroni correction was used for analysis among the 4 groups. *P<0.00833. NS, not significant. Data in E through G are shown as dot plots. dnNRSF‐Tg indicates dominant‐negative form of neuron‐restrictive silencer factor transgenic mice; ANOVA, analysis of variance; cont, control.

**Figure 2.**
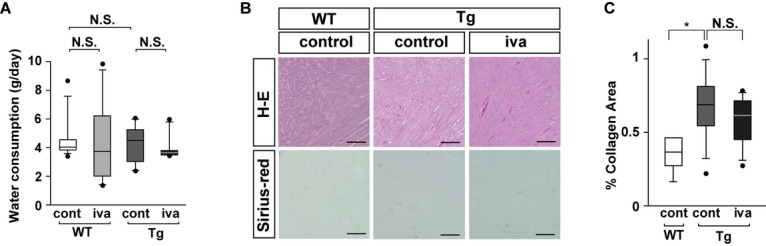
Effects of ivabradine (iva) on the water consumption and histology in dnNRSF‐Tg (Tg) mice. A, Water consumption (g/day) in 12‐week‐old WT and Tg mice, with or without iva (n=8 for untreated [cont] WT mice, n=6 for WT mice treated with iva, n=6 for untreated (cont) Tg mice, n=6 for Tg mice treated with iva). Kruskal–Wallis nonparametric ANOVA followed by the Bonferroni correction was used for analysis among the 4 groups. B, Histology of WT and Tg hearts from 20‐week‐old mice treated with or without iva: H‐E, Hematoxylin‐Eosin staining; scale bars, 100 μm. C, Graphs show the percentage of collagen area in untreated (cont) WT mice, Tg mice treated without (cont) or with iva (n=5 for untreated WT mice, n=9 for untreated Tg mice, n=7 for Tg mice treated with iva). Kruskal–Wallis nonparametric ANOVA followed by the Bonferroni correction was used for the analysis among the 3 groups. *P<0.0166. All data are shown as box plots. NS indicates not significant; WT, wild type; cont, control; dnNRSF‐Tg, dominant‐negative form of neuron‐restrictive silencer factor transgenic mice; ANOVA, analysis of variance.

**Figure 3.**
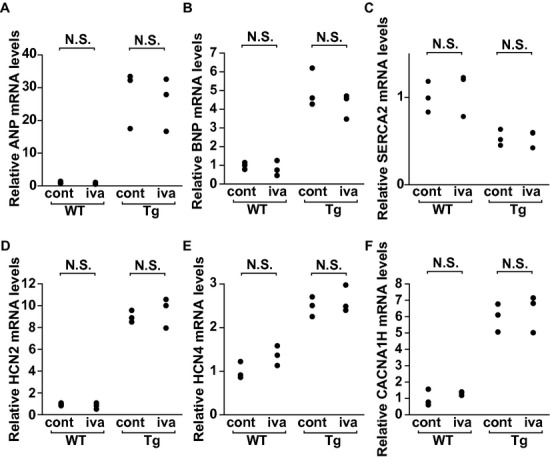
Effects of ivabradine (iva) on the gene expression in dnNRSF‐Tg (Tg) mice. A through F, Relative levels of ANP (A), BNP (B), SERCA2 (C), HCN2 (D), HCN4 (E), and CACNA1H (F) mRNA in hearts from 20‐week‐old WT and Tg mice treated with or without iva. n=3 in each group. Mice treated without iva are indicated as cont. Kruskal–Wallis nonparametric ANOVA followed by the Bonferroni correction was used for analysis among the 4 groups. All data are shown as dot plots. NS indicates not significant; ANP, atrial natriuretic peptide; cont, control; WT, wild type; dnNRSF‐Tg, dominant‐negative form of neuron‐restrictive silencer factor in transgenic mice; BNP, brain natriuretic peptide; SERCA2, sarcoplasmic/endoplasmic reticulum calcium ATPase 2; HCN2, hyperpolarization‐activated cyclic nucleotide‐gated channel 2; CACNA1H, calcium channel, voltage‐dependent, T type, alpha 1H subunit; ANOVA, analysis of variance.

**Figure 4.**
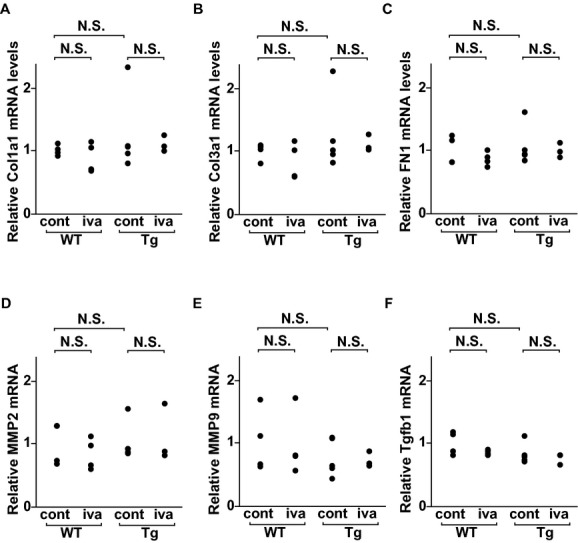
Effects of ivabradine (iva) on the fibrosis‐related genes expression in dnNRSF‐Tg (Tg) mice. A through F, Relative levels of Col1a1 (A), Col3a1 (B), FN1 (C), MMP2 (D), MMP9 (E), and Tgfb1 (F) mRNA in hearts from 20‐week‐old WT and Tg mice treated with or without iva. n=4 for WT without iva, n=4 for WT with iva, n=5 for Tg without iva, and n=3 for Tg with iva. Mice treated without iva are indicated as cont. Kruskal–Wallis nonparametric ANOVA followed by the Bonferroni correction was used for the analysis. All data are shown as dot plots. NS indicates not significant; dnNRSF‐Tg, dominant‐negative form of neuron‐restrictive silencer factor transgenic mice; ANOVA, analysis of variance; WT, wild type; cont, control; Col1a1, collagen type1 α1; Col3a1, collagen type3 α 1; FN1, fibronectin 1; MMP2, matrix metallopeptidase 2, MMP9, matrix metallopeptidase 9; Tgfb1, transforming growth factor‐ β 1.

We previously showed that dnNRSF‐Tg mice develop progressive cardiomyopathy and begin to die from ventricular tachyarrhythmias at about 8 weeks of age.^9^ In dnNRSF‐Tg hearts, *HCN2* and *HCN4*, 2 genes encoding HCN channels and transcriptional targets of NRSF/REST, were upregulated, and there was a corresponding increase in I_f_ amplitude in the isolated ventricular myocytes. By contrast, no stable I_f_ currents were recorded in adult ventricular myocytes from the WT littermates' hearts.^9^ To determine the role played by HCN channels in the development of malignant arrhythmias and sudden death, and to assess the potential therapeutic effect of HCN channel blockade in dnNRSF‐Tg mice, we administered ivabradine, a specific HCN channel blocker, beginning when the mice were 8 weeks of age. Initially, we confirmed that ivabradine significantly blocked I_f_ currents in ventricular myocytes from dnNRSF‐Tg mice (Figure 1A and 1B). In Figure 1A, we applied 3 μmol/L ivabradine, which is close to the half maximal inhibitory concentration (IC_50_) for all HCN channels.^19^ Although at a dose of 7 mg/kg per day, ivabradine reduced heart rate in WT mice, it did not significantly affect heart rate in dnNRSF‐Tg mice, whose basal heart rates were slower than those of WT mice (Figure 1C and Table 1). Despite having no effect on heart rate, ivabradine significantly improved the survival rate among dnNRSF‐Tg mice (Figure 1D). Blood pressures, body weights, heart‐to‐body weight ratios, and lung‐to‐body weight ratios did not differ between the control and ivabradine groups in either genotype (Table 1 and Figure 1E through 1G). On the other hand, heart‐to‐body weight ratios were significantly higher in dnNRSF‐Tg mice than WT mice, as described previously.^9^ At a dose of 7 mg/kg per day, ivabradine did not affect water consumption in either genotype, which is consistent with an earlier report (Figure 2A).^12^ Echocardiographic and histological parameters, including the percentage of fibrosis area, showed no significant differences between untreated and ivabradine‐treated dnNRSF‐Tg mice (Table 1 and Figure 2B and 2C). Likewise, hemodynamic parameters measured during cardiac catheterization did not significantly differ between the 2 groups of dnNRSF‐Tg mice (Table 1). By contrast, echocardiography and cardiac catheterization showed that, as compared to untreated WT mice, left ventricular systolic function was disturbed and the percentage of fibrotic area was significantly increased in untreated dnNRSF‐Tg mice, as described previously (Figure 2C and Table 1).^9^ Consistent with those findings, there was no significant difference in the expression of the cardiac stress marker genes *ANP*,* BNP*, and *SERCA2* between the untreated and ivabradine‐treated groups within either genotype, whereas the expression of these genes differed between untreated WT mice and untreated dnNRSF‐Tg mice as described previously (Figure 3A through 3C).^9,20–21^ Moreover, the mRNA expression levels of *HCN2* and *HCN4*, as well as *CACNA1H*, which encodes the T‐type calcium channel, did not differ between untreated and ivabradine‐treated mice within either genotype (Figure 3D through 3F). The ventricular expression levels of *HCN2*,* HCN4*, and *CACNA1H* mRNA were higher in dnNRSF‐Tg mice than WT mice as described previously (Figure 3D through 3F).^9^ Expression of the fibrosis‐related genes collagen type1 α1 (*Col1a1*), collagen type3 α 1 (*Col3a1*), fibronectin 1 (*FN1*), matrix metallopeptidase 2 (*MMP2*), matrix metallopeptidase 9 (*MMP9*), and transforming growth factor‐β 1 (*Tgfb1*) did not differ between WT and dnNRSF‐Tg mice and was not affected by ivabradine treatment in either genotype (Figure 4A through 4F). All of these data suggest that ivabradine directly suppresses sudden death in dnNRSF‐Tg mice without affecting heart rate or cardiac structure or function.

**Table 1.** Hemodynamic Parameters in 20‐Week‐Old WT and dnNRSF‐Tg Mice Treated With or Without Ivabradine

	WT		dnNRSF‐Tg	
	Control	Iva	Control	Iva
Blood pressure, mm Hg	105.1±3.7	100.4±6.6	100.2±1.1	98.3±3.5
Heart rate, /min	671±36.6	541±37.7	576±25.8	550±15.1
Echocardiographic data				
LVDd, mm	3.3±0.18	3.13±0.19	4.11±0.14*	4.04±0.08*
LVDs, mm	1.65±0.13	1.4±0.12	2.87±0.13*	2.67±0.06*
IVST, mm	0.89±0.03	0.83±0.04	0.77±0.03	0.76±0.03
PWT, mm	0.93±0.04	0.87±0.03	0.82±0.04	0.79±0.04
FS, %	50.3±1.8	54.0±1.2	30.5±1.2*	33.6±1.3*
Hemodynamic data				
LVSP, mm Hg	102.9±1.9	101.0±0.2	92.6±1.1	93.1±1.9
LVEDP, mm Hg	4.0±1.1	4.9±1.3	6.4±1.4	6.0±1.0
dP/dt, mm Hg/s	11 729.6±200.5	11 779.6±462.8	5754.7±626.5	6580.3±445.6
−dP/dt, mm Hg/s	5429.5±179.0	5584.1±462.8	4306.0±227.0	4500.8±474.6

Values are means±SEM. Numbers of mice tested for blood pressure and heart rate are 9 for untreated control WT, 5 for WT with ivabradine, 10 for untreated dnNRSF‐Tg, and 6 for dnNRSF‐Tg with ivabradine. Numbers of mice tested for echocardiography are 8 for untreated control WT, 3 for WT with ivabradine, 14 for untreated dnNRSF‐Tg, and 13 for dnNRSF‐Tg with ivabradine. Numbers of mice tested for hemodynamic analysis are 3 for each group. Kruskal–Wallis nonparametric ANOVA followed by the Bonferroni correction was used for analysis among the 4 groups. WT indicates wild type; dnNRSF‐Tg, dominant‐negative form of neuron‐restrictive silencer factor transgenic mice; Control, untreated control mice; Iva, mice treated with ivabradine; LVDd, left ventricular diastolic dimension; LVDs, left ventricular systolic dimension; IVST, interventricular septum thickness; PWT, posterior wall thickness; FS, fractional shortening; LVSP, left ventricular systolic pressure; LVEDP, left ventricular end diastolic pressure; dP/dt, first derivative of pressure; ANOVA, analysis of variance.

* *P*<0.00833 vs untreated wild type.

### Ivabradine Suppresses Arrhythmicity in dnNRSF‐Tg Mice

**Figure 5.**
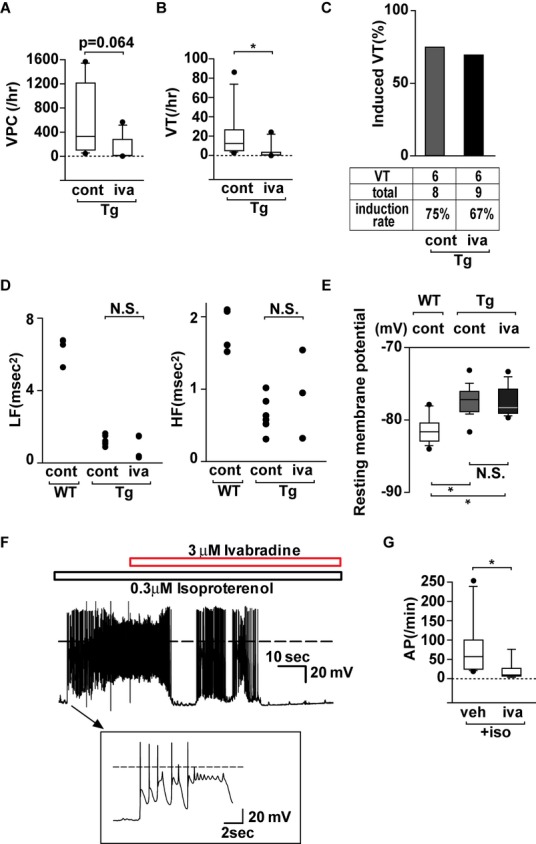
Ivabradine (iva) reduces arrhythmicity in dnNRSF‐Tg (Tg) hearts. A and B, Numbers of PVCs (A) and VTs (B) recorded using a telemetry system in Tg mice treated with or without iva. Data are shown as box plots. Mann–Whitney test was used for analysis. *P<0.05 (n=7 for Tg without iva, 6 for Tg with iva). C, Frequency of mice with inducible VTs during intracardiac electrophysiology studies among Tg mice treated for 12 weeks with or without iva. VT, numbers of mice with inducible VT; total, total numbers of mice tested. D, Average power of the low frequency (LF) and high frequency (HF) components of HRVs recorded over a 24‐hour period in untreated WT and Tg mice treated with or without iva. Mice treated without iva are indicated as cont. n=4 for WT without iva, n=6 for Tg without iva, n=4 for Tg with iva. Kruskal–Wallis nonparametric ANOVA followed by Bonferroni correction was used for the analysis. Data are shown as dot plots. E, Average resting membrane potentials recorded from ventricular myocytes isolated from 20‐week‐old untreated WT and Tg mice treated with or without iva: Kruskal–Wallis nonparametric ANOVA followed by the Bonferroni correction was used for the analysis. NS, not significant. *P<0.0166 vs WT (n=12 for untreated WT, 12 for untreated Tg, 14 for Tg with iva). Mice treated without iva are indicated as cont. Data are shown as box plots. F, Representative traces showing that ivabradine (3 μmol/L) reduces the frequency of spontaneous action potentials in the presence of isoproterenol (0.3 μmol/L) are shown. Arrows show larger pictures of action potentials. G, Graphs show numbers of spontaneous action potentials in the presence of isoproterenol (0.3 μmol/L) in ventricular myocytes from dnNRSF‐Tg in the absence (veh) or presence of iva. Shown are the numbers of spontaneous action potentials (AP/min) occurring in the presence of isoproterenol (0.3 μmol/L) during the second minute after addition of iva or vehicle (veh). Data are shown as box plots. Mann–Whitney test was used for the analysis (n=6 for control and 5 for iva). *P<0.05. NS indicates not significant; dnNRSF‐Tg indicates dominant‐negative form of neuron‐restrictive silencer factor transgenic mice; PVC, premature ventricular contraction; VT, ventricular tachycardia; ANOVA, analysis of variance; WT, wild type; cont, control; HRV, heart rate variability.

We next used a telemetric monitoring system to examine the effects of ivabradine on electrocardiographic parameters in dnNRSF‐Tg mice. We found that ivabradine tended to suppress the number of premature ventricular contractions (PVCs) in dnNRSF‐Tg hearts (Figure 5A). More importantly, it dramatically reduced the number of episodes of ventricular tachycardia (VT) (Figure 5B). To elucidate the mechanism by which ivabradine suppressed arrhythmias in dnNRSF‐Tg mice, we evaluated its effect on arrhythmogenic re‐entrant substrates by carrying out an in vivo intracardiac electrophysiological analysis in dnNRSF‐Tg mice.^9,16^ We found that dnNRSF‐Tg mice were highly susceptible to induction of VT, as reported previously^9^ (Figure 5C), and that ivabradine did not reduce their susceptibility (Figure 5C), indicating that ivabradine does not modulate the arrhythmogenic re‐entrant substrates observed in dnNRSF‐Tg ventricles. We also assessed the effect of ivabradine on autonomic nerve activity by analyzing HRV, as imbalanced autonomic nerve activity can trigger ventricular arrhythmias in failing hearts.^22–23^ As we previously reported,^21^ HRV was diminished in dnNRSF‐Tg mice, and was unaffected by ivabradine (Figure 5D). All of these results indicate that ivabradine suppresses malignant arrhythmias without affecting re‐entrant substrates or autonomic imbalance in dnNRSF‐Tg mice. This suggests ivabradine acts directly to suppress abnormal impulse formation caused by abnormally enhanced automaticity or triggered activity.

### Ivabradine Reduces the Frequency of Spontaneous Action Potential in Cardiac Ventricular Myocytes From dnNRSF‐Tg Mice

We also evaluated the effects of ivabradine on the abnormal impulse formation seen in ventricular myocytes from dnNRSF‐Tg mice. We first examined the effects of long‐term treatment with ivabradine on the electrophysiological properties of myocytes isolated from dnNRSF‐Tg hearts. As we showed previously,^14^ the resting membrane potential was somewhat depolarized in ventricular myocytes isolated from dnNRSF‐Tg hearts, as compared to WT hearts, which was largely attributed to the decrease in density of inward‐rectifier K^+^ current.^24^ Ivabradine did not significantly affect the resting membrane potential in dnNRSF‐Tg myocytes (Figure 5E).

We previously reported that in the presence of isoproterenol, ventricular myocytes isolated from dnNRSF‐Tg hearts showed early after‐depolarizations and spontaneous action potentials, but myocytes from WT hearts did not, demonstrating the higher susceptibility of dnNRSF‐Tg myocytes to β‐adrenergically–induced arrhythmias.^9,24^ Moreover, it is known that increasing cyclic adenosine monophosphate (cAMP) levels through β‐adrenergic stimulation causes I_f_ currents to become faster and larger in amplitude than the currents elicited under unstimulated conditions.^2,6^ We therefore assessed the effect of ivabradine on the response of dnNRSF‐Tg myocytes to β‐adrenergic stimulation. In dnNRSF‐Tg ventricular myocytes exposed to isoproterenol, ivabradine tended to reduce the occurrence of spontaneous action potentials (Figure 5F and 5G), which supports our idea that increased ventricular expression of HCN channels leads to an abnormal increase in automaticity that contributes to arrhythmogenesis in dnNRSF‐Tg mice.

### Increased Expression of HCN2 in Ventricular Myocytes Promotes Susceptibility to Arrhythmias Induced by Chronic Isoproterenol Treatment

**Figure 6.**
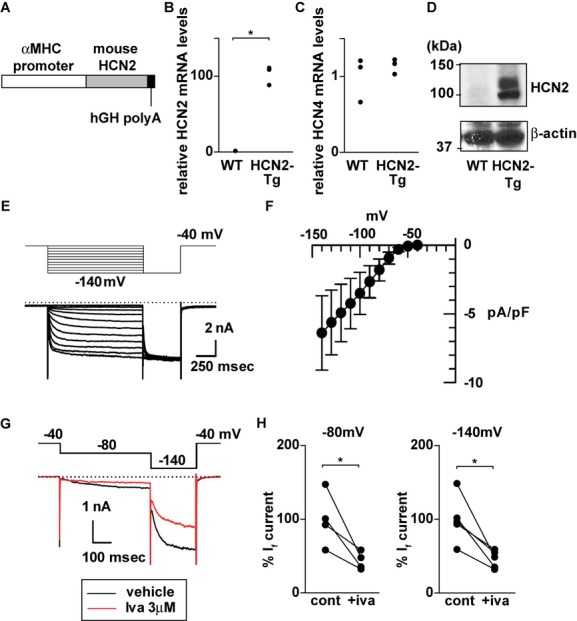
Generation of cardiac‐specific HCN2 transgenic mice. A, Scheme of the construct for HCN2 transgenic mouse. hGH, human growth hormone. B and C, Relative levels of HCN2 (B) and HCN4 (C) mRNA in hearts from 12‐week‐old WT and HCN2‐Tg mice. The Mann–Whitney test was used for the analysis. *P<0.05 vs WT. n=3 in each group. Data are shown as dot plots. D, Representative Western blots for HCN2 and β‐actin in ventricular myocytes from WT and HCN2‐Tg mice. E, Representative I_f_ currents recorded in ventricular myocytes from HCN2‐Tg mice. Inward‐rectifier K^+^ current was suppressed by 0.5 mmol/L BaCl_2_. F, Current‐voltage relationship for I_f_ in ventricular myocytes from HCN2‐Tg mice. The amplitudes of time‐dependent components activated by hyperpolarizing pulses were normalized by cellular capacitance (n=5). G, Effect of 3 μmol/L ivabradine (iva) (red line; 2 minutes after the application of iva) or vehicle (cont) on I_f_ in ventricular myocytes from HCN2‐Tg mice. H, Graphs show the suppressive effect of iva on I_f_ amplitude at −80 mV (left panel, n=4 each) and −140 mV (right panel, n=5 each) in ventricular myocytes from HCN2‐Tg mice. The mean relative I_f_ amplitudes (%) in the absence of iva were assigned a value of 100. The Wilcoxon signed‐rank test was used for the analysis. *P<0.05 vs cont. HCN2‐Tg indicates hyperpolarization‐activated cyclic nucleotide‐gated channel 2 transgenic mice; WT, wild type; cont, control; αMHC, α‐myosin heavy chain.

To further assess the role of induced HCN channel expression in increased cardiac arrythmicity, we generated transgenic mice exhibiting specific cardiac expression of HCN2 driven by the α‐MHC promoter (HCN2‐Tg) (Figure 6A). In HCN2‐Tg mice, ventricular HCN2 mRNA levels were significantly higher than in their WT littermates (Figure 6B), whereas ventricular HCN4 mRNA levels were similar in the 2 genotypes (Figure 6C). In addition, HCN2‐Tg mice showed a 50‐fold increase in the expression of HCN2 protein, as compared to WT mice (Figure 6D). We also confirmed the presence of I_f_ currents in ventricular myocytes from HCN2‐Tg mice, and that they were inhibited by 3 μmol/L ivabradine (Figure 6E through 6H). By contrast, I_f_ currents were not detected in ventricular myocytes from the WT littermates (data not shown), as reported previously.^9^ The amplitudes of I_f_ currents in HCN2‐Tg ventricular myocytes were ≈1.5 times larger than in dnNRSF‐Tg myocytes (Figure 6F). ^9^

**Figure 7.**
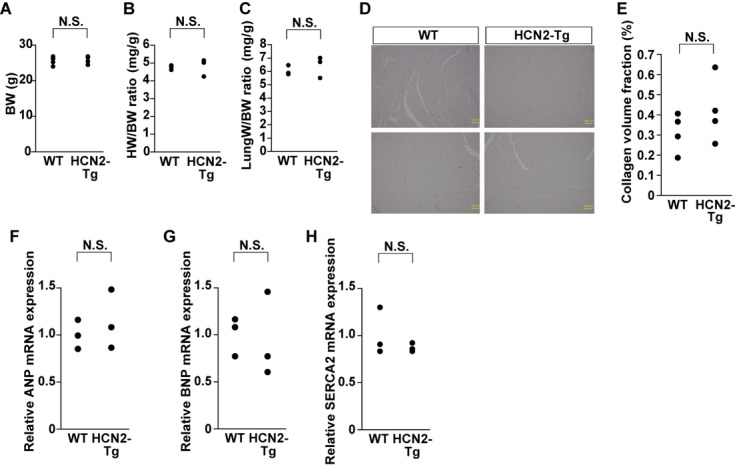
Features of cardiac‐specific HCN2‐Tg mice. A through C, Body weights (BW) (A), heart weight‐to‐body weight ratios (HW/BW) (B) and lung‐to‐body weight ratios (LW/BW) (C) in 12‐week‐old WT and HCN2‐Tg mice are shown as dot plots (for BWs, n=4 for each group; for HW/BW ratios, n=3 for each group; and for LW/BW ratios, n=3 for each group). The Mann–Whitney test was used for the comparison between WT and HCN2‐Tg. NS, not significant. D, Histology of WT and HCN2‐Tg hearts from 12‐week‐old mice: Sirius‐red staining. Magnification, ×400; scale bars, 100 μm. E, Graph showing the the percentage of collagen area in WT and HCN2‐Tg mice (n=4 for each group). The Mann–Whitney test was used for the analysis. NS, not significant. Data are shown as dot plots. F through H, Relative levels of ANP (F), BNP (G), and SERCA2 (H) mRNA in hearts from 12‐week‐old WT and HCN2‐Tg mice (n=3 in each group). The Mann–Whitney test was used for the analysis. NS, not significant. Data are shown as dot plots in F through H. HCN2‐Tg indicates hyperpolarization‐activated cyclic nucleotide‐gated channel 2 transgenic mice; WT, wild type.

HNC2‐Tg mice raised under normal conditions were viable and fertile, and their body weights, heart‐to‐body weight ratios, and lung‐to‐body weight ratios did not differ from their WT littermates (Figure 7A through 7C). In addition, there were no significant differences in the echocardiographic, hemodynamic, and histological findings between the 2 groups, except that HCN2‐Tg mice showed significantly faster heart rates than control WT mice (Figure 7D and 7E, and Table 2). Consistent with those results, there were no significant differences in the expression of the cardiac stress marker genes *ANP*,* BNP*, and *SERCA2* between the 2 groups (Figure 7F through 7H). Taken together, these findings demonstrate that increased ventricular expression of HCN2 and the resultant increase in the I_f_ current by themselves are not sufficient to significantly alter cardiac structure or systolic function.

**Table 2.** Hemodynamic Parameters in 8‐Week‐Old WT and HCN2‐Tg Mice

	WT	HCN2‐Tg
Blood pressure, mm Hg	103.3±2.9	104.8±2.3
Heart rate, /min	527±29.5	659±22.2*
Echocardiographic data		
LVDd, mm	2.53±0.12	2.53±0.08
LVDs, mm	1.15±0.10	1.08±0.07
IVST, mm	0.73±0.02	0.68±0.02
PWT, mm	0.72±0.03	0.73±0.02
FS, %	53.8±2.0	56.7±2.8

Values are means±SEM. Numbers of mice tested are 6 for each genotype. The Mann–Whitney test was used for comparison between WT and HCN2‐Tg. WT indicates wild type; HCN2‐Tg, hyperpolarization‐activated cyclic nucleotide‐gated channel 2 transgenic mice; LVDd, left ventricular diastolic dimension; LVDs, left ventricular systolic dimension; IVST, interventricular septum thickness; PWT, posterior wall thickness; FS, fractional shortening.

* *P*<0.05 vs control wild‐type littermates.

**Figure 8.**
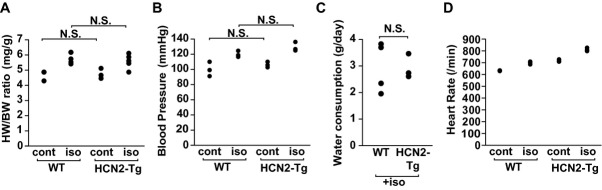
Effects of β‐adrenergic stimulation in HCN2‐Tg mice. A and B, HW/BW ratios (A) and blood pressures (B) in 24‐week‐old WT and HCN2‐Tg mice, with or without 1 week of isoproterenol (iso) administration (15 mg/kg per day; subcutaneous infusion): *P<0.05. (for HW/BW ratios, n=3 for WT without iso, n=5 for WT with iso, n=3 for Tg without iso, n=5 for Tg with iso; for blood pressure, n=3 in each group). Mice treated without iso are indicated as cont. Kruskal–Wallis nonparametric ANOVA followed by the Bonferroni correction was used for analysis among the 4 groups. NS, not significant. All data are shown as dot plots. C, Water consumption (g/day) in 24‐week‐old WT and Tg mice treated for 1 week with iso (15 mg/kg per day; subcutaneous infusion) (n=4 for WT mice, n=3 for Tg mice). The Mann–Whitney test was used for the analysis. NS, not significant. Data are shown as dot plots. D, Heart rate assessed by ambulatory electrocardiography in 24‐week‐old WT and HCN2‐Tg mice, with or without 1 week of isoproterenol (iso) administration (15 mg/kg per day; subcutaneous infusion): Kruskal–Wallis nonparametric ANOVA followed by the Bonferroni correction was used for analysis among the 4 groups. n=3 for each group. Data are shown as dot plots. HCN2‐Tg indicates hyperpolarization‐activated cyclic nucleotide‐gated channel 2 transgenic mice; WT, wild type; cont, control; HW/BW, heart weight‐to‐body weight ratios; ANOVA, analysis of variance.

**Figure 9.**
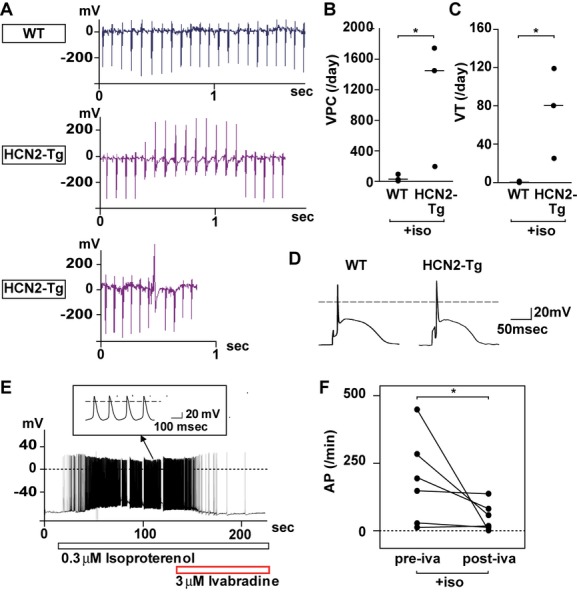
β‐adrenergic stimulation‐induced ventricular arrhythmias in HCN2‐Tg hearts. A, Representative ECG traces showing sinus rhythm in WT mice treated with isoproterenol (upper panel) and ventricular tachycardia (VT) with atrioventricular dissociation (middle panel) and isolated PVC (lower panel) in HCN2‐Tg mice treated with isoproterenol. B and C, Numbers of isolated PVCs (B) and VTs (C) recorded using a telemetry system in WT and HCN2‐Tg mice treated with isoproterenol (iso). Mann–Whitney test was used for the analysis. *P<0.05 (n=3 for each group). D, Representative traces of action potentials recorded in ventricular myocytes isolated from WT and HCN2‐Tg hearts. E, Representative traces of spontaneous action potentials in ventricular myocytes from HCN2‐Tg hearts recorded in the presence of 0.3 μmol/L isoproterenol, to which 3 μmol/L ivabradine was subsequently added, as indicated. Arrows show larger pictures of action potentials. F, Graph showing the numbers of spontaneous action potentials (AP) in the presence of 0.3 μmol/L isoproterenol (iso) in ventricular myocytes from HCN2‐Tg mice with or without 3 μmol/L ivabradine (iva). The Wilcoxon signed‐rank test was used for the analysis. Shown are the numbers of action potentials occurring during the minute just before (pre‐iva) and the second minute after (post‐iva) addition of ivabradine. *P<0.05 (n=6). HCN2‐Tg indicates hyperpolarization‐activated cyclic nucleotide‐gated channel 2 transgenic mice; WT, wild type; PVC, premature ventricular contraction.

We also examined the effect of β‐adrenergic stimulation on HCN2‐Tg mice. When we used an osmotic mini pump to subcutaneously administer isoproterenol to HCN2‐Tg and WT mice for a week, both genotypes showed comparable cardiac hypertrophic responses, as assessed from heart‐to‐body weight ratios (Figure 8A). Systolic function evaluated based on echocardiographic findings and blood pressure did not differ between the 2 genotypes treated with isoproterenol (Figure 8B and Table 3). Water intake was also not altered in either genotype treated with isoproterenol (Figure 8C). Heart rate in HCN2‐Tg treated with isoproterenol was faster than those in WT mice treated with isoproterenol (Figure 8D), whereas the percentage increase induced by isoproterenol in heart rate was comparable between 2 genotypes (10.1% increase in WT mice versus 13.0% increase in HCN2‐Tg mice). By contrast, continuous ECG monitoring during the administration of isoproterenol showed that PVC and VT frequencies were significantly higher in HCN2‐Tg than WT mice (Figure 9A through 9C). Thus increased expression of ventricular HCN2 channels appears to promote susceptibility to isoproterenol‐induced cardiac arrhythmias without affecting cardiac structure or function.

**Table 3.** Echocardiographic Data in 24‐Week‐Old WT and HCN2‐Tg Mice Treated With Isoproterenol for 1 Week

	WT+Iso	HCN2‐Tg+Iso
LVDd, mm	3.09±0.18	3.14±0.21
LVDs, mm	1.68±0.21	1.66±0.33
FS, %	46.4±4.1	49.6±7.8

Values are means±SEM. Numbers of mice tested are n=5 for WT treated with isoproterenol and 4 for HCN2‐Tg treated with isoproterenol. The Mann–Whitney test was used for comparison between WT+Iso and HCN2‐Tg+Iso.WT indicates wild type; HCN2‐Tg, hyperpolarization‐activated cyclic nucleotide‐gated channel 2 transgenic mice; Iso, isoproterenol; LVDd, left ventricular diastolic dimension; LVDs, left ventricular systolic dimension; FS, fractional shortening.

When we then analyzed action potentials in ventricular myocytes isolated from HCN2‐Tg and WT hearts, we found no apparent difference between the groups (Figure 9D). We then assessed the response of HCN2‐Tg myocytes to β‐adrenergic stimulation. After the application of 0.3 μmol/L isoproterenol, the resting membrane potential of ventricular myocytes isolated from HCN2‐Tg hearts slowly depolarized, and started to fire spontaneously (Figure 9E), whereas such spontaneous activity was not induced in the myocytes from WT hearts. Ivabradine blocked the isoproterenol‐related occurrence of spontaneous action potentials in ventricular myocytes from HCN2‐Tg mice (Figure 9E and 9F). These data support our hypothesis that increased HCN channel expression in ventricular myocytes potentially increases the susceptibility to arrhythmias under sympathetic stimulation.

## Discussion

In healthy adult hearts, expression of HCN channels is restricted to the sinoatrial node and other parts of the conduction system. However, their expression is induced in the failing and hypertrophied ventricular myocardium.^6–7^ In earlier studies, we showed that a transcriptional repressor, NRSF, negatively regulates the expression of the *HCN2* and *HCN4* genes and that a transcriptional activator, MEF2, positively regulates *HCN4* expression in cardiac myocytes.^9,25–26^ Indeed, expression of both *HCN2* and *HCN4* is upregulated in dnNRSF‐Tg mice, leading to an increase in the I_f_ current in ventricular myocytes. Such increases in I_f_ may predispose ventricular myocytes to enhanced automaticity, which can in turn trigger malignant arrhythmias. To clarify the contribution made by increased expression of HCN channels to the development of arrhythmias in dnNRSF‐Tg mice, a useful model of heart failure with lethal arrhythmias, we tested the effects of ivabradine, a HCN channel blocker, on survival and arrhythmicity. We found that ivabradine significantly reduced the incidence of sudden death and malignant arrhythmias among dnNRSF‐Tg mice without significantly reducing heart rate or affecting cardiac function, most likely by suppressing an increase in automaticity. We also confirmed the increased susceptibility to arrhythmias induced by β‐adrenergic stimulation in mice specifically overexpressing the cardiac HCN2 channel. Collectively, our findings provide the first evidence that increased expression of HCN channels contributes to the increased arrhythmicity seen in failing hearts in vivo, and suggest that HCN channel blockade may represent a new and effective means of preventing sudden arrhythmic death in patients with heart failure.

There is now compelling evidence that I_f_ currents play a key role in cardiac pacemaking.^27^ Consistent with that idea, an I_f_ blocker, ivabradine, can reduce heart rate without exerting negative inotropic or vasodilatory effects.^28^ As basal heart rate is an independent risk factor for mortality in patients with heart failure,^29–30^ it had been thought that an agent that selectively lowered heart rate could improve outcomes in patients with heart failure. As would be expected, results from a recent clinical trial, the Systolic Heart failure treatment with the I_f_ inhibitor ivabradine Trial (SHIFT), showed that among heart failure patients with resting heart rates of 70 beats/min or higher, the negative chronotropic effect of ivabradine significantly improved their outcomes.^31^ In addition to this human clinical trial, several studies using animal models revealed the beneficial effects of ivabradine‐induced heart rate reduction on cardiac structure and electrical remodeling.^32^ In our present study, to evaluate the direct effects of ivabradine on the arrhythmicity of ventricular myocytes and the survival of dnNRSF‐Tg mice, while avoiding the effects of heart rate reduction, we used a relatively low dose of ivabradine (7 mg/kg per day), which did not significantly affect heart rates in dnNRSF‐Tg mice, though it did reduce heart rates by around 20% in WT mice (Table 1). This likely reflects the fact that basal heart rates were significantly lower in dnNRSF‐Tg mice than WT mice (Table 1). From these findings, we conclude that the beneficial effects of ivabradine on arrhythmicity and survival in dnNRSF‐Tg mice largely reflect direct suppression of arrhythmicity in ventricular myocytes, independent of heart rate, although the possibility that ivabradine prevents deaths due to congestive heart failure in some dnNRSF‐Tg mice cannot be excluded completely. The observation that HCN channels and I_f_ currents are upregulated in the ventricular myocardium of hypertrophied and failing hearts in both rodent models and humans^6–7^ suggests that, in some clinical settings, ivabradine may improve the outcomes of patients with heart failure by both reducing heart rate and acting via mechanisms independent of heart rate reduction.

Our results showing that ivabradine did not prevent cardiac dysfunction and structural remodeling in dnNSRF‐Tg mice under conditions in which heart rate was not significantly reduced by the drug, suggest that increased I_f_ amplitude in ventricular myocytes does not contribute substantially to cardiac structural remodeling and depressed systolic function. Our finding that cardiac‐specific overexpression of *HCN2* and the corresponding increase in I_f_ amplitude did not affect cardiac structure or systolic function support this notion. These results suggest the beneficial effects of ivabradine on cardiac structural remodeling and dysfunction observed in several animal models and in human patients with heart failure largely depend on its effect on heart rate mediated through suppression of I_f_ currents in the sinus node.^31–32^ This is consistent with a recent subanalysis of the morBidity‐mortality EvAlUaTion of the I_f_ inhibitor ivabradine in patients with CAD and left ventricULar dysfunction (BEAUTIFUL) study, in which the impact of ivabradine treatment on cardiovascular outcome in patients with stable coronary artery disease and left ventricular systolic dysfunction was tested. Echocardiography revealed that the reduction in the left ventricular systolic volume index seen with ivabradine treatment was related to the degree of heart rate reduction.^33–34^ In addition, a subanalysis of the SHIFT study, in which the effects of ivabradine treatment on outcome in heart failure patients with resting heart rates >70 beats/min were studied, also showed that the improvement in left ventricular systolic function seen with ivabradine treatment was associated with a decrease in heart rate.^31,35^

Sudden cardiac death is generally caused by ventricular tachycardia or fibrillation. These arrhythmias occur due to the simultaneous presence of abnormal impulse initiation (the trigger), which results from either enhanced automaticity or triggered activity, and a preexisting arrhythmogenic substrates for the initiation and maintenance of reentry.^36–37^ In dnNRSF‐Tg mice, in vivo electrophysiological analysis showed that ivabradine failed to attenuate the increase in susceptibility to induced arrhythmias dependent on the presence of arrhythmogenic substrates. Ivabradine did not prevent the generation of arrhythmogenic substrates in dnNRSF‐Tg hearts, which is consistent with the results showing that ivabradine does not affect cardiac structural remodeling, including the fibrosis and cardiac dysfunction seen in dnNRSF‐Tg mice. This suggests that increased ventricular expression of HCN channels contributes to the generation of arrhythmogenic triggers but not to the generation of arrhythmogenic substrates. The fact that cardiac‐specific overexpression of HCN2 did not alter the normal cardiac structure and systolic function supports this finding. These observations are also consistent with recent reports showing that mice lacking both cardiac HCN2 and HCN4 developed cardiac hypertrophy in response to pressure overload induced by transaortic constriction (TAC), like WT mice do, whereas ventricular myocytes isolated from the double knockout mice subjected to TAC showed weaker proarrhythmogenic parameters than those from WT mice subjected to TAC.^38^ The potential contribution made by ventricular HCN channels to the repolarization process was also apparent in ventricular myocytes from knockout mice lacking HCN3 or HCN1,^39^ which can contribute to an increase in arrhythmogenic triggers. Still, it is possible that ivabradine also inhibits the generation of arrhythmogenic substrates, such as interstitial fibrosis, thereby suppressing the susceptibility to induced arrhythmias under conditions where it reduces heart rates, as stated above. Further studies in other animal models of cardiac disease and in humans with heart failure would be of great interest.

The autonomic control of heart rate and sinoatrial node activity is largely mediated by the I_f_ current, which is specifically modulated by intracellular cAMP.^40^ Thus, upregulation of HCN channels may enhance the arrhythmicity of failing ventricular myocytes exposed to β‐adrenergic stimulation. Consistent with this notion, ventricular myocytes from dnNRSF‐Tg mice showed abnormal spontaneous action potentials when exposed to isoproterenol,^14^ and this effect was attenuated by ivabradine (Figure 5F and 5G). Similarly, ventricular myocytes from HCN2‐Tg mice exhibited spontaneous action potentials in the presence of isoproterenol, and that effect too was inhibited by ivabradine (Figure 9E and 9F). By contrast, this response was not seen in myocytes from WT hearts. These results suggest that upregulation of HCN channels, and the resultant increase in I_f_ currents predispose failing hearts to arrhythmias in a setting of enhanced sympathetic drive. Indeed, in an earlier study, we showed that activation of sympathetic activity contributes to sudden death in dnNRSF‐Tg mice;^14^ moreover, our present findings show that HCN2‐Tg mice are highly susceptible to isoproterenol‐induced arrhythmias.

Ivabradine was developed for clinical use in the treatment of stable angina pectoris as a heart rate‐lowering agent and was recently shown to significantly improve outcomes in patients with heart failure and elevated heart rate (70 beats/min or above).^28,31,40^ Although in the SHIFT study sudden cardiac death did not appear to be affected by ivabradine,^31^ this finding may be attributed to the effect of background treatment with a β‐blocker (used in 89% of patients), which effectively prevents sudden cardiac death.^41–42^ A recently published post hoc subgroup analysis of SHIFT study showed that reduction of cardiovascular death by ivabradine among subjects without β‐blocker treatment was not statistically significant.^43^ However, the numbers of subjects without β‐blocker treatment is much smaller than the overall sample size to detect effects of ivabradine. Further investigation will be needed to determine whether ivabradine will prevent sudden cardiac death in heart failure patients who cannot tolerate a β‐blocker.

## Disclosure

This work was supported, in part, by a grant from Institut de Recherches Internationales Servier (to Dr Kuwahara).
